# Isolation and Characterization of Earthworm Peptides with Neuroprotective Effects in Parkinson’s Disease Models

**DOI:** 10.3390/molecules30091952

**Published:** 2025-04-28

**Authors:** Guangyu Shi, Yikao Hu, Xiaolin Bai, Xun Liao

**Affiliations:** 1 Chengdu Institute of Biology, Chinese Academy of Sciences, Chengdu 610041, China; shigy@cib.ac.cn (G.S.); huyk@cib.ac.cn (Y.H.); baixl@cib.ac.cn (X.B.); 2 University of Chinese Academy of Sciences, Beijing 100049, China

**Keywords:** earthworm, neuroprotection, PINK1 agonistic activity, *PINK1^B9^* flies, anti-Parkinson’s disease

## Abstract

The aim of this study was to identify peptides from *Lumbricus terrestris* with neuroprotective effects. Two peptides (GYSFTTTAER and AVFPSIVGR) isolated from earthworms improved cell viability of the SH-SY5Y human neuroblastoma cell line treated with 1-methyl-4-phenyl-1,2,3-tetrahydropyridinehydrochloride (MPP^+^), a commonly used model of Parkinson’s disease (PD). Both peptides increased the mitochondrial membrane potential and upregulated the mRNAs of mitophagy regulators PINK1 and Parkin in the MPP^+^-damaged cells. The in vitro assay and molecular docking indicated that both peptides exhibited moderate PINK1 agonistic activity. Furthermore, GYSFTTTAER and AVFPSIVGR extended the lifespan, improved locomotor behavior, and raised the ATP and dopamine levels at all ages in *PINK1^B9^* mutant flies, a PD model characterized by loss-of-function of PINK1. These findings suggest that earthworm-derived peptides possess anti-neurodegenerative properties and hold potential for the development of health products and therapeutic agents for PD.

## 1. Introduction

Earthworm is used in traditional Chinese medicine as an animal-derived remedy. Its protein content and amino acid composition are better than those of fish, cow milk, and soybean meal [1]. In addition, compared to general plant protein, earthworm protein exhibits a closer resemblance to human protein in terms of its amino acid pattern. Moreover, its essential amino acid utilization rate is higher in the body and is more readily assimilated by the human system. Therefore, it is used as a supplement in animal feed and human food. Earthworm has been recorded in the Chinese medical classics of “*Compendium of Materia Medica*” for treating thrombus, hypertension, asthma, convulsion, and cardiovascular diseases [2,3]. Pharmacological studies revealed that earthworm possesses a multitude of biological functions, encompassing antibacterial and anti-tumor effects, relieving asthma, and promoting wound healing [4,5,6,7]. In addition, extracts of earthworm also promote the regeneration of nerve cells and expedite the recovery of nerve function following injury [8,9]. Peptides have become essential active ingredients in animal drugs due to their remarkable therapeutic effects at low doses coupled with minimal side effects, making them particularly valuable for disease prevention and treatment. Research indicates that lumbricusin (RNRRWCIDQQA), an antimicrobial peptide derived from earthworm, promotes SH-SY5Y cells proliferation and inhibits apoptosis and decreased viability induced by 1-methyl-4-phenyl-1,2,3-tetrahydropyridinehydrochloride (MPP^+^) [10]. In addition, lumbricusin analog 5 (LumA5, QLICWRRFR) has been shown to significantly reduce the expression of lip-induced inflammatory enzymes in vitro and in vivo, resulting in improved viability of SH-SY5Y cells, and is considered as a potential drug for the treatment of a variety of inflammatory diseases including Parkinson’s disease [11].

Parkinson’s disease (PD) is the second most common neurodegenerative disease, and it is characterized by involuntary shaking, muscle rigidity, and the progressive loss of dopaminergic neurons [12] Currently, PD cannot be diagnosed before the onset of clinical symptoms, which also complicates the treatment of the disease [13]. PD is caused by multiple factors, including genetic and environmental factors. Its pathogenesis is associated with oxidative stress, mitochondrial dysfunction, and abnormal protein aggregation [14]. At present, the medicines used to treat PD include levodopa, resagiline, and silagiline, which cannot fundamentally prevent the degeneration of dopaminergic neurons and usually have serious side effects when taken for long periods of time [15]. Therefore, it is necessary to explore new anti-PD drugs with high activity and small side effects.

PTEN-induced putative kinase 1 (PINK1) plays a pivotal role in regulating mitochondrial quality control and regulating the selective elimination of damaged mitochondria via autophagy [16]. Inhibiting PINK1 kinase activity leads to the early onset of familial PD [17]. Therefore, the development of medicines that can restore normal levels of mitochondrial autophagy may help stop the neurodegenerative process and fight against PD. Interestingly, certain natural products, such as celastrol, salidroside, and resveratrol, have been found to exert their therapeutic effects on PD by upregulating the expression of PINK1 [18,19,20,21]. However, peptides from natural products such as PINK1 agonists have not yet been reported. Simultaneously, the development of PINK1 agonists is still in its nascent stage and necessitates further investigation.

In this study, our objective was to isolate and identify active peptides in earthworm by using column chromatography and LC-MS/MS. The ability to rescue the SH-SY5Y cells injured by 1-methyl-4-phenyl-1,2,3,6-tetrahydropyridinehydrochloride (MPP^+^) was utilized for demonstrating the neuroprotective effect of earthworm peptides. Mitochondrial damage and autophagy activity were assessed after MPP^+^ exposure, with or without peptide treatment. In addition, the role of peptides in the anti-PD function of earthworm was investigated by assessing their PINK1 agonistic activity, and molecular docking analysis elucidated the peptide–PINK1 interaction mechanism including affinity and docking site. The *PINK1^B9^* flies genetic model of PD was used to evaluate the anti-PD effects, including longevity, climbing ability, and dopamine and ATP levels. The obtained results provide confirmation of the neuroprotective properties and potential anti-PD effects of earthworm peptides, thereby establishing a solid scientific basis for the utilization of earthworm as a therapeutic agent for the treatment of Parkinson’s disease.

## 2. Results

### 2.1. Isolation and Identification of Earthworm Peptides

**Figure 1:**
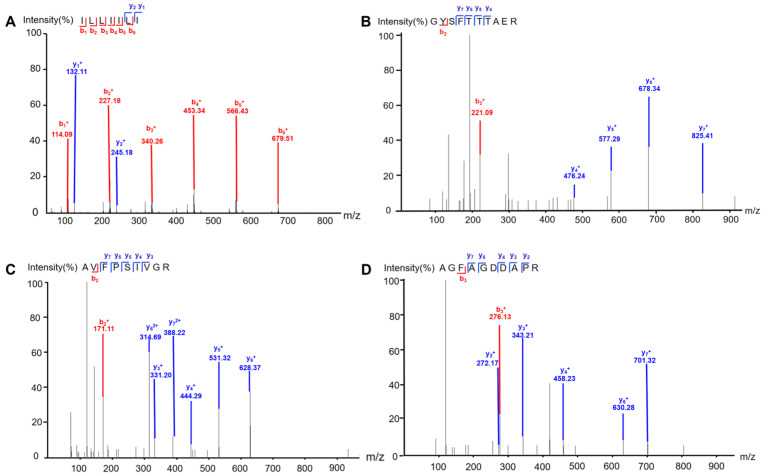
Tandem mass spectrometry (LC-MS/MS) spectra of the identified peptides. (**A**) ILLIILI; (**B**) GYSFTTTAER; (**C**) AVFPSIVGR; (**D**) AGFAGDDAPR. “b_1_^+^–b_6_^+^” represent the b ions the fragmentation of which occurred from N-terminal of the peptide, while b_1_ means one amino acid, b_2_ two amino acids, and so on. “y_1_^+^–y_7_^+^” represent the y ions the fragmentation of which occurred from the C-terminal of the peptide, while y_1_ means one amino acid, y_2_ two amino acids, and so on. y_6_^2+^ and y_7_^2+^ represent the double-charged ions of six and seven amino acids fragmented from C-terminal of the peptide, respectively.

Extract of earthworm was separated by column chromatography guided by the protective effect on MPP^+^-induced SH-SY5Y cells to afford four peptides. The peptides were identified by comparing MS/MS data with the protein database of earthworm downloaded from Uniport (https://www.uniprot.org/uniprotkb?query=earthworm) (accessed on 19 March 2025). As it is generally believed that XCorr > 1 has a high degree of credibility, four peptides were identified as ILLIILI, GYSFTTTAER, AVFPSIVGR, and AGFAGDDAPR (Figure 1 and Table S1). Further, we synthesized these four peptides to confirm their structures. It is noted that MS/MS alone cannot distinguish I from L; however, by comparing fragmentation ion information determined by MS/MS against earthworm protein sequences, ILLIILI can be identified. Interestingly, we found that there were a large number of peptides with C-terminus Arg or Lys residue. This might have resulted from the digestive enzymes present in earthworm released during the drying and extraction process.

### 2.2. Neuroprotective Activity Bioassays

#### 2.2.1. Neuroprotective Effects of Peptides in MPP^+^-Induced Injury of SH-SY5Y Cells

**Figure 2:**
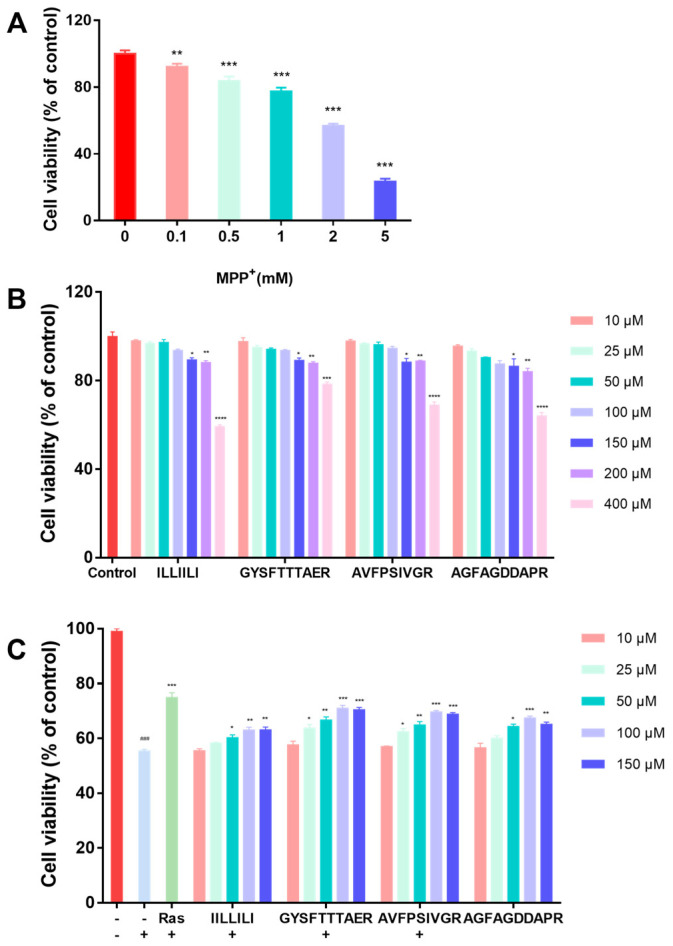
Cytotoxic effect of MPP^+^ and neuroprotective effects of the peptides on MPP^+^-damaged SH-SY5Y cells. (**A**) Effect of MPP^+^ on cell viability of SH-SY5Y cells. (**B**) Cytotoxic effects of peptides on SH-SY5Y cells. (**C**) Neuroprotective effects of peptides on MPP^+^ -induced SH-SY5Y cells. Cell viability was evaluated using the CCK-8 assay. “+” represents the presence of the material indicated to the left of the X-axis MPP^+^; “-” represents the absence of the material indicated to the left of the X-axis. Data from the three-times-repeated independent experiments are expressed as the mean ± SD (n = 3 in each group; ### *p* < 0.001, compared with the control group; * *p* < 0.05, ** *p* < 0.01, *** *p* < 0.001 and **** *p* < 0.0001 compared with the model group). Rasagiline (Ras) was used as a positive control.

MPP^+^-induced mitochondrial dysfunction and oxidative stress are involved in the pathogenesis of PD [22]. First, the MPP^+^ concentration was determined to establish a PD cell model. Cells were incubated with different concentrations of MPP^+^ for 24 h by comparing the degree of cellular damage induced by a range of concentrations. As shown in Figure 2A, the cell viability was significantly reduced in a dose-dependent manner. The survival rate of cells exposed to 2 mM MPP^+^ was 56.25%, which satisfied the modeling requirements. Consequently, this dosage was employed for subsequent experiments. Second, peptides (10, 25, 50, 100, 150, 200, and 400 μM) were tested for cytotoxicity against SH-SY5Y cells, and the results are shown in Figure 2B, wherein the safe concentration range for these peptides in SH-SY5Y cells was determined to be between 10 and 150 μM. Finally, peptides with concentrations ranging from 10 μM to 150 μM were selected to further investigate the neuroprotective effects. The results, as shown in Figure 2C, revealed that cell viability was restored to 70.08% and 68.77% upon treatment with GYSFTTTAER (100 μM) and AVFPSIVGR (100 μM), indicating that they displayed significant neuroprotective effects. Especially, GYSFTTTAER at a concentration of 100 μM showed the best neuroprotective effect, which was similar to the positive control rasagiline.

#### 2.2.2. Morphological Analyses of SH-SY5Y Cells

**Figure 3:**
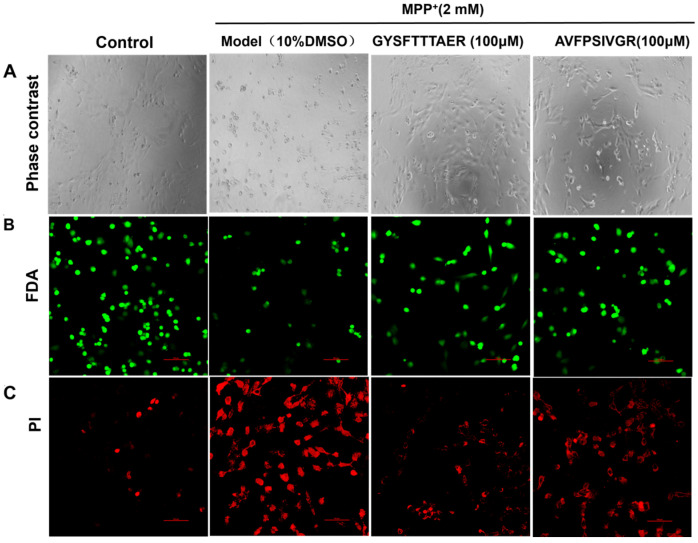
Morphological changes of SH-SY5Y cells under different conditions. (**A**) Observation under phase-contrast microscope. (**B**) SH-SY5Y cells were stained with FDA and visualized using a confocal laser scanning microscope. (**C**) SH-SY5Y cells were stained with PI and visualized using a confocal laser scanning microscope. The FDA and PI images were taken under different microscopic fields.

Due to GYSFTTTAER (100 μM) and AVFPSIVGR (100 μM) showing better neuroprotection, we examined their effects on the morphology of MPP^+^-injured SH-SY5Y cells. As shown in Figure 3A, the cells treated with MPP^+^ demonstrated pronounced cellular shrinkage when compared to the untreated cells. However, the morphological changes of the cells were significantly improved following treatment with GYSFTTTAER (100 μM) and AVFPSIVGR (100 μM). The cells were stained with FDA/PI to visualize cell damage, with the results depicted in Figure 3B,C, wherein the number of live cells stained by FDA increased, and the number of damaged cells stainable by PI decreased compared with the model group. The results indicated that both GYSFTTTAER and AVFPSIVGR exhibited significant protective effects against MPP^+^-induced apoptosis in SH-SY5Y cells, with GYSFTTTAER demonstrating superior efficacy compared to AVFPSIVGR.

### 2.3. Effects of Earthworm Peptides on the Mitochondrial Damage and Mitophagy in MPP^+^-Treated SH-SY5Y Cells

**Figure 4:**
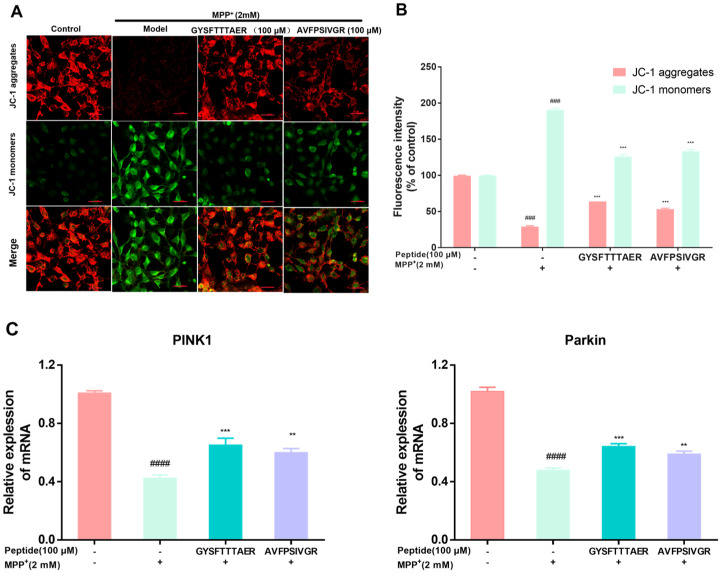
Effect of earthworm peptides on mitochondrial membrane potential and the mRNA levels of PINK1 and Parkin in SH-SY5Y cells. (**A**) The effect of mitochondrial membrane potential caused (MPP) by MPP^+^ exposure was strongly restored by earthworm peptides, as analyzed by JC-1. Scale bar is 100 μm. (**B**) Fluorescence quantification by ImageJ software, Version 1.54p. (**C**) The mRNA levels of PINK1 and Parkin in MPP^+^-treated SH-SY5Y cells. The data are presented as the mean value ± SD (n = 3 in each group; ### *p* < 0.001, and #### *p* < 0.0001, compared with the control group; ** *p* < 0.01, and *** *p* < 0.001, compared with the model group).

Mitochondrial membrane potential (MMP) reduction is a key indicator of mitochondrial damage. High MMP allows JC-1 to aggregate in the mitochondrial matrix, producing red fluorescence, while low MMP results in JC-1 existing as monomers, generating green fluorescence [23]. Figure 4A,B showed that SH-SY5Y cells exhibited decreased red and enhanced green fluorescence compared to the control cells, indicating MMP reduction, while the 100 µM peptide pre-treatment group enhanced the red fluorescence and decreased the green fluorescence compared to the model cells, suggesting MMP elevation. Furthermore, we investigated the protective effect of two peptides on mitophagy induction during MPP^+^ treatment. PINK1 and Parkin are crucial regulators of mitophagy [24]. The MPP^+^-damaged cells obviously reduced the mRNA levels of PINK1 and Parkin compared to the control cells. Conversely, the pre-treatment with two peptides significantly elevated the PINK1 and Parkin mRNA levels (Figure 4C). These findings indicated that both peptides exert significant neuroprotective effects by activating mitophagy.

### 2.4. PINK1 Agonistic Activity of Earthworm Peptides

The results indicated that both GYSFTTTAER and AVFPSIVGR exhibited significant protective effects against MPP^+^-induced apoptosis in SH-SY5Y cells, with GYSFTTTAER demonstrating superior efficacy compared to AVFPSIVGR. The results of PINK1 activity screening of peptides are shown in Table 1. When the concentration was 100 μM, the excitability levels of PINK1 by GYSFTTTAER and AVFPSIVGR were 52.46 ± 0.96% and 50.35 ± 0.72%, respectively. The calculated EC_50_ levels were 76.77 ± 1.3 μM and 88.5 ± 0.97 μM, respectively, with moderate PINK1 agonistic activity.

### 2.5. Molecular Docking of Peptides and PINK1

**Figure 5:**
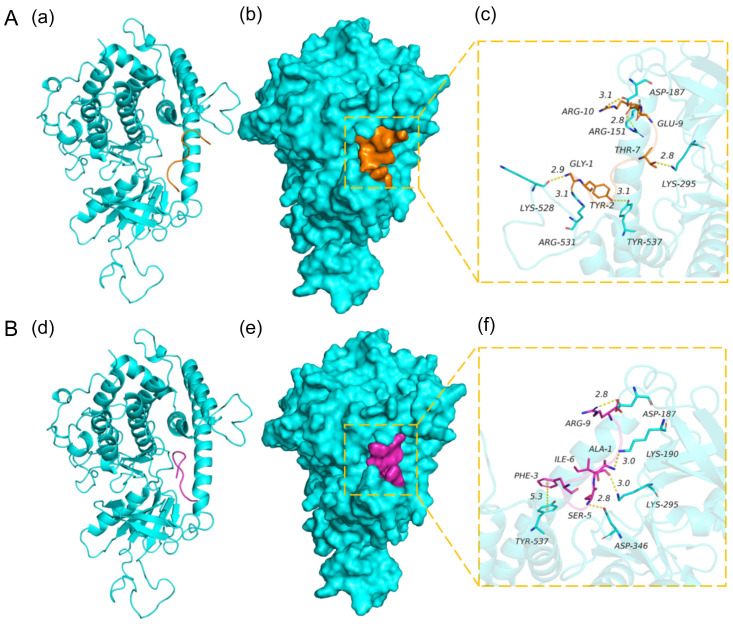
(**A**). The binding mode of PINK1 with GYSFTTTAER. (**a**) The 3D structure of the complex. (**b**) The electrostatic surface of the protein. (**c**) The detail binding mode of the complex. The backbone of protein was rendered in the tube and colored in cyan. The peptide is rendered in orange. (**B**). The binding mode of PINK1 with AVFPSIVGR. (**d**) The 3D structure of the complex. (**e**) The electrostatic surface of the protein. (**f**) The detail binding mode of the complex. Peptide is rendered in red. The main chain of the protein is displayed in a tubular form to more clearly observe the secondary structure of the protein and the binding position of the peptide to the protein.

The interaction between ligands and enzymes is commonly investigated through molecular docking, which involves the analysis of docking energies, binding sites, and critical residues. The peptides GYSFTTTAER and AVFPSIVGR were individually subjected to molecular docking with PINK1, as shown in Figure 5. The results of the molecular docking demonstrated a strong binding affinity between the peptides and target proteins, exhibiting excellent compatibility. The binding energy scores were −10.365 kcal/mol and −9.328 kcal/mol for GYSFTTTAER and AVFPSIVGR, respectively. The Pymol software (Version 2.1.0) was utilized for visualizing the complexes formed by the docked peptides and proteins, while also providing access to the binding patterns of these peptides and proteins. The amino acid residues within peptides and protein pockets can be distinctly observed based on their binding patterns. As shown in Figure 4A, GYSFTTTAER can form hydrogen-bonding interactions with Lys-295, Tyr-537, Arg-531, and Lys-528 in the active site of PINK1, as well as two ionic bonds (salt bridges) with Asp-187 and Arg-151, which are essential for stabilizing the peptide molecule [25]. As shown in Figure 4B, AVFPSIVGR was able to form hydrogen-bonding interactions with Lys-190, Lys-295, Tyr-537, and Asp-346 at the active site of the PINK1, as well as ionic bonding (salt bridges) with Asp-187, and these interactions could effectively promote the formation of stable complexes between the peptide and the protein. In addition, Phe-3 of the peptide can also form a strong hydrophobic interaction with Tyr-537, thereby making an indispensable contribution to the molecular stabilization. Moreover, according to Figure 5B (e), we observed a strong affinity between AVFPSIVGR and PINK1, indicating their favorable interaction for the formation of a stable complex. In addition, both peptides demonstrated significant correlation with protein targets, with GYSFTTTAER exhibiting superior performance.

### 2.6. Neuroprotective Effect of Peptides on PINK1^B9^ Flies

Due to their superior PINK1 agonist activity demonstrated in vitro, GYSFTTTAER and AVFPSIVGR were selected for further investigation of their neuroprotective effects on *PINK1^B9^* flies.

#### 2.6.1. Lifespan of PINK1^B9^ Flies Was Extended by Earthworm Peptides

**Figure 6:**
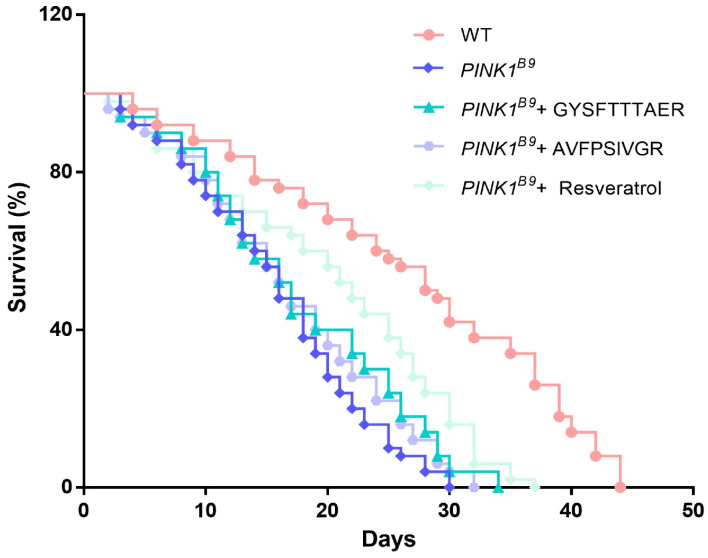
Lifespan of WT flies, *PINK1^B9^* flies, *PINK1^B9^* flies treated with 0.1 mM of peptides, and *PINK1^B9^* flies treated with 1 mM of resveratrol.

Resveratrol, a well-established antioxidant with significant protective effects against pathologic markers associated with PD, exhibits potential for therapeutic application in PD patients [26]. Studies have shown that in *PINK1^B9^* flies, high concentrations (1 mM) of resveratrol were effective in extending the lifespan of *PINK1^B9^* flies but had no significant effect at low concentrations (0.2 mM) [27]. As shown in Figure 6, the lifespan of *PINK1^B9^* flies was shortened compared to WT. Conversely, GYSFTTTAER (0.1 mM), AVFPSIVGR (0.1 mM), and resveratrol (1 mM) extended the lifespan of *PINK1^B9^* by 13.33%, 6.67%, and 23.33% respectively. The results indicated that earthworm peptides can extend the lifespan of *PINK1^B9^* flies; the effect is not as good as resveratrol.

#### 2.6.2. Locomotor Ability of PINK1^B9^ Flies Was Improved by Earthworm Peptides

**Figure 7:**
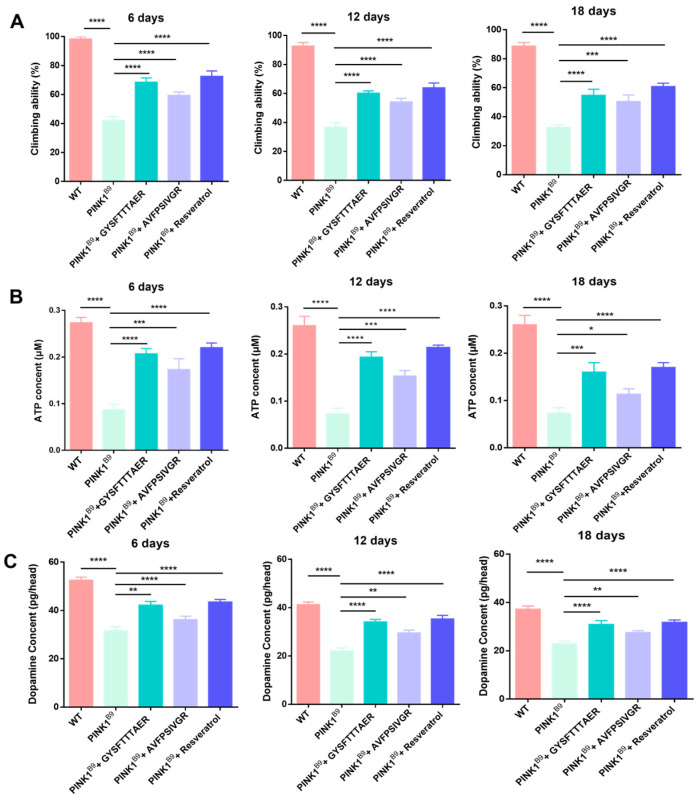
Climbing abilities of WT flies, *PINK1^B9^* flies, *PINK1^B9^* flies treated with 0.1 mM of peptides, and *PINK1^B9^* flies treated with 1 mM of resveratrol at 6, 12, and 18 days (**A**). The ATP levels of WT flies, *PINK1^B9^* flies, and *PINK1^B9^* flies treated 1 mM of resveratrol. (**B**) Dopamine behavior of WT flies, *PINK1^B9^* flies, *PINK1^B9^* flies treated with 0.1 mM of peptides, and *PINK1^B9^* flies treated with 1 mM of resveratrol at 6, 12, and 18 days (**C**). Data from three independent experiments were expressed as means ± SD (n = 3 in each group; * *p* < 0.05, ** *p* < 0.01, *** *p* < 0.001, and **** *p* < 0.0001).

As depicted in Figure 7A, the climbing experiment revealed a significant reduction in climbing activity among *PINK1^B9^* flies, with percentages of 42.13% at 6 days old, 36.77% at 12 days old, and 32.70% at 18 days old. These findings indicate that *PINK1^B9^* flies have comparatively weaker climbing ability than the WT flies. In comparison, the addition of 0.1 mM GYSFTTTAER significantly enhanced the locomotor ability of *PINK1^B9^* flies, resulting in improvements to 68.52%, 60.13%, and 54.79% at the three age stages, respectively. Similarly, treatment with 0.1 mM AVFPSIVGR also led to improved locomotor ability in *PINK1^B9^* flies, with percentages reaching 59.42%, 54.81%, and 50.7% at the three age stages. The present study demonstrates that earthworm peptides significantly enhance the climbing ability of *PINK1^B9^* flies across all age groups.

#### 2.6.3. ATP Content Increment

The presence of PINK1 is crucial for maintaining mitochondrial homeostasis, and the absence of PINK1 leads to a reduction in ATP levels [28]. As depicted in Figure 7B, the ATP content of *PINK1^B9^* flies was significantly reduced with age. Importantly, treatment with 0.1 mM of GYSFTTTAER or AVFPSIVGR resulted in significant increases in ATP levels at 6, 12, and 18 days.

#### 2.6.4. Dopamine Content Increment

The significant pathological features of *PINK1^B9^* flies were the notable reduction in dopamine levels within the brain [29]. As shown in Figure 7C, the dopamine content of *PINK1^B9^* flies decreased largely compared to that of WT flies, and it gradually decreased with age. After treatment with 0.1 mM of GYSFTTTAER or AVFPSIVGR, the level of dopamine was significantly increased at 6, 12, and 18 days, respectively.

## 3. Materials and Methods

### 3.1. Materials and Reagent

The earthworms (*Lumbricus terrestris*) were purchased from Beijing Tongrentang Co., Ltd. (Chengdu, China). They were dried by the supplier through sun-drying and stored at 4 °C. The shelf life is one year. Rasagiline was purchased from Aladdin Biochemical Technology Co., Ltd. (Shanghai, China). Resveratrol was purchased from Pusi Biotechnology Co., Ltd. (Chengdu, China). The SH-SY5Y cells were obtained from the National Collection of Authenticated Cell Cultures (Shanghai, China). The MPP^+^ was purchased from Sigma-Aldrich Co., Ltd. (Shanghai, China). Dulbecco’s modified Eagle’s medium/Ham’s F-12 (DMEM/F12), fetal bovine serum (FBS), and phosphate-buffered saline (PBS) were purchased from Biological Industries (Kibbutz Beit HaEmek, Israel). Fluorescein diacetate (FDA), propidium iodide (PI) staining and mitochondrial membrane potential test kit (JC-1) were purchased from Beijing Solarbio Science & Technology Co., Ltd. (Beijing, China). Cell Counting Kit-8 (CCK-8) was purchased from Bimake Co., Ltd. (Shanghai, China). PrimeScript RT reagent kit and *PerfectStart* Green PCR Master Mix were purchased from TransGen Biotech (Beijing, China). PINK1 (86.30 U/mL) was expressed and purified according to a previous report [30]. The Kinase-Lumi™ Luminescent Kinase Assay Kit and ATP Assay Kit were purchased from Beyotime Biotechnology Co., Ltd. (Wuhan, China). The Insect dopamine (DA) ELISA Kit was purchased from Jiangsu Meimian Industrial Co., Ltd. (Yancheng, Jiangsu, China). HPLC-grade methanol was supplied by XLJ (Yunnan, China). Ultrapure water used in the experiment was prepared using a UP water purification (18.25 MΩ) system (Chengdu, China). Unless otherwise specified, chemicals and solvents were purchased from Kelong Chemical Reagents Factory (Chengdu, China).

### 3.2. Isolation and Identification of Earthworm Peptides

#### 3.2.1. Crude Earthworm Extract

Firstly, the dried earthworm (1.0 kg) was powdered and then extracted with 0.9% sodium chloride solution (10 L) at a temperature of 100 °C for 1 h. After filtration, the residue was subjected to secondary extraction refluxing at 100 °C for 30 min with the addition of a 0.9% sodium chloride solution (8 L), and enzymes were not added during the whole extraction process. Finally, the filtrates were pooled and evaporated under reduced pressure to obtain the crude extract with an extraction rate of 4.2%; the neuroprotective effect on MPP^+^ induced SH-SY5Y cells was 60.38%.

#### 3.2.2. Peptide Purification

The crude extract was dissolved in 50% methanol, followed by centrifugation to obtain the supernatant. The supernatant was then loaded onto a Sephadex LH-20 gel filtration column (2.5 × 200 cm) and eluted by 50% methanol with thin-layer chromatography (TLC) as the preliminary guide for fractionation. Three fractions were obtained with the neuroprotective effect on MPP^+^-induced SH-SY5Y cells of 59.85%, 60.74%, and 62.54%, respectively. The most potent fraction was further purified by column chromatography on RP-18 (3.0 × 30 cm) eluted with gradient water–methanol to give two fractions, and their neuroprotective effects on MPP^+^-induced SH-SY5Y cells were 62.87% and 64.63%, respectively.

#### 3.2.3. Identification of the Peptides

The fraction with 64.63% neuroprotective effect was subjected to LC-MS/MS analysis with a Q Exactive™ Hybrid Quadrupole-Orbitrap™ mass spectrometer (Thermo Fisher Scientific Inc., Waltham, MA, USA). Before LC-MS/MS analysis, the sample was reduced and alkylated with dithiothreitol and iodoacetamide, respectively, and then dissolved in 0.1% (v/v) formic acid (FA). An Acclaim PepMap RPLC C18 (3 µm, 75 µm×150 mm Thermo Fisher Scientific Inc.) column was used for LC separation, and the mobile phase consisted of A (0.1% formic acid in water) and B (0.1% formic acid in acetonitrile). The gradient elution procedure was as follows: 3–63 min, 0–8% B; 63–68 min, 8–28% B; 68–70 min, 28–95% B; 70–72 min, 95% B; the flow rate was 0.25 μL/min. The analytes were detected by setting the spray voltage and capillary temperature at 2.2 kV and 270 °C, respectively. Full scan resolution was set to 70000 at *m/z* 400–1600, and the mass range was adjusted to *m/z* 350–1800. The raw data were imported into Proteome Discoverer 10.0 software (Thermo Fisher Scientific Inc., USA) for peptide identification, and the peptides were identified by comparing MS/MS data with the protein database of earthworm downloaded from Uniport (https://www.uniprot.org/uniprotkb?query=earthworm) (accessed on 19 March 2025).

### 3.3. Peptides Synthesis

The identified peptides were synthesized using a solid-phase synthesis method provided by Sangon Biotech Co., Ltd. (Shanghai, China). The counterion in the synthesized peptide sample was trifluoroacetic acid with a concentration of less than 2%. The purity of peptides was determined to be greater than 95% by HPLC, and the sequences of peptides were verified by LC-MS/MS using the de novo method.

### 3.4. Neuroprotective Activity Bioassays

#### 3.4.1. Cell Cultures and Cell Viabilities

The SH-SY5Y human neuroblastoma cell line is extensively employed as a prominent cellular model for PD [31]. The neuroprotective effect of earthworm peptides against the MPP^+^-damaged SH-SHY5Y cell model was evaluated using the CCK-8 method. First, the SH-SHY5Y cells were cultured in DMEM/F12 medium supplemented with 10% fetal bovine serum (FBS) and 1% penicillin–streptomycin, and they were maintained at a temperature of 37 °C with a CO_2_ concentration of 5% in a humidified atmosphere. The SH-SHY5Y cells were seeded into 96-well plates at a density of 1 × 10^4^ cells per well, followed by incubation at 37 °C for 24 h. Subsequently, the initial medium was carefully aspirated. The peptide samples were dissolved in DMSO to prepare a high-concentration stock solution and then diluted to the required concentration (10, 25, 50, 100, and 150 μM), prepared using a medium, where they were subsequently introduced into the well and incubated at 37 °C for a duration of 2 h. Then, the cells were treated with MPP^+^ (2 mM) per well for an additional 24 h. Finally, we added 10 μL of CCK-8 solution to each well and incubated the mixture at 37 °C for 1 h.

Rasagiline was used as the positive control. Following the completion of the experiment, the 96-well plate was extracted, and the absorbance was measured at 450nm by the MULTISKAN GO spectrophotometric plate reader (Thermo Scientific, Waltham, MA, USA). The data were analyzed using GraphPad Prism, version 6.0.

#### 3.4.2. Morphological Analyses and Fluorescence Staining of SH-SY5Y Cells

The SH-SY5Y cells were seeded into 6-well plates at a density of 1 × 10^5^ cells per well of medium for a duration of 24 h. The peptides were separately added to the administered and modeled groups, followed by incubation at 37 °C for a duration of 2 h. The subsequent addition of MPP^+^ (2 mM) was followed by an incubation period lasting 24 h. The cell morphology was examined using fluorescence microscopy (Leica dmi1, Wetzlar, Germany). The cells were washed with PBS and subsequently stained with 10 μg/mL FDA and 5 μg/mL PI. Following a 30 min incubation, the staining solution was aspirated, and the cells were rinsed with PBS. Finally, cell images were acquired using a confocal microscope (Nikon Eclipse Ti-2, Tokyo, Japan).

### 3.5. Measurement of Mitochondrial Membrane Potential

SH-SY5Y cells were seeded into 6-well plates at a density of 2.5 × 10^5^ cells/well in 2.0 mL of medium for 24 h and pretreated with or in the absence of earthworm peptides (100 μM) for 2 h, followed by induction with MPP^+^ (2 mM) for another 24 h. The medium was discarded, and 1ml JC-1 working solution was added; then, the mixture was incubated at 37 °C for 20 min and washed with JC-1 staining buffer twice, and 2mL cell culture solution was then added. The changes were observed using a confocal laser scanning microscope (Nikon Eclipse Ti-2, Japan).

### 3.6. Quantitative qPCR

Total RNA was extracted with a TransZol Up RNA extraction kit (TransGen Biotech, Beijing, China) according to the manufacturer’s instructions. Reverse transcription was performed with a PrimeScript RT reagent kit. Real-time PCR was performed in a CFX 96 Connect™ Optics Module using *PerfectStart* Green PCR Master Mix (TransGen Biotech, Beijing, China). Aliquots of cDNA were analyzed by PCR using primer sets specific to PINK1, Parkin, and β-actin (as a control). Primers were as follows: PINK1-F: 5′-GGCCCAGATGTCGTCTCAAA-3′, PINK1-R: 5′-TCCCGGCAGAAAACGAACC-3′; Parkin-F: 5′-TCCCAACTCCCTGATTAAAGAGC-3′, Parkin-R: 5′-ACGCCTTCCAATGTAGATCCC-3′, ACTB-F: 5′-CACCATTGGCAATGAGCGGTTC-3′, ACTB-R: 5′-AGGTCTTTGCGGATGTCCACGT-3′. Data were analyzed according to the 2^−ΔΔct^ method, as previously described.

### 3.7. Determination of the Agonistic Activity of PINK1

The PINK1 activity assay was conducted with certain modifications based on the described by [32]. Ubiquitin is a potent substrate for PINK1, which catalyzes the phosphorylation of ubiquitin while consuming ATP [33]. During the reaction, the consumption of ATP is associated with PINK1 activity. The quantification of kinase activity was performed using chemiluminescence to determine the residual amount of ATP in solution following the kinase reaction, enabling calculation of the agonistic activity of peptide samples against PINK1 kinase [34]. The enzymatic reaction was conducted on a white 96-well plate, with all samples dissolved in a buffer solution containing 50 mM Tris-HCl (pH = 7.5), 0.1 mM EDTA, 10 mM MgCl_2_, and 4% 2-hydroxy-1-ethanethiol. Subsequently, 10 μL of peptide sample, 10 μL of PINK1 (6.8 μM), 10 μL of ubiquitin (0.002 pg/mL), and 20 μL of ATP (10 μM) were added to the plate for incubation at a temperature of 25 °C for a duration of ten minutes. In addition, the blank control group replaces the peptide with a buffer solution. The resveratrol was employed as a positive control. The amount of ATP consumed was detected using the Kinase-Lumi™ Luminescent Kinase Assay Kit. The PINK1 agonistic rate was calculated by the following equation:

(1) $$PINK1\;agonistic\;rate\;(\%)=(1-\frac{A-B}{C-D})\times 100\%$$

where A represents the absorbance of the sample group consisting of the sample, PINK1, ubiquitin, and ATP; B represents the absorbance of the sample control group consisting of the sample, PINK1, and ubiquitin; C represents the absorbance of the control group consisting of PINK1, ubiquitin, and ATP; D represents the absorbance of the blank control group consisting of PINK1 and ubiquitin. The EC_50_ of each peptide was calculated using GraphPad Prism, version 10.0.2 software.

### 3.8. Molecular Docking Study

Molecular docking of the peptides was performed by using Schrödinger Maestro, version 2019.01 software. The 3D structures of ligands were constructed in ChemBioDraw Ultra 14.0, and further optimized by ChemBio3D Ultra 14.0.0.117 (CambridgeSoft, USA). The crystal structure of PINK1 protein was found according to its amino acid sequence using the homology modeling website Swiss-Model for building (https://swissmodel.expasy.org/, the template: 7 mp8, 98.89%) (accessed on 19 March 2025). PINK1 protein structure was processed on the Maestro 11.9 platform. The Schrodinger protein preparation wizard was used to treat the protein, including removing the crystal water (crystal water is water molecules that bind to the surface or inside of protein molecules during the protein crystallization process), adding the missing hydrogen atom, and repairing the missing bond information [25,35]. The peptide ligands and PINK1 macromolecule were subjected to molecular docking using the flexible docking tool provided by Autodock, version 1.2.5 software. The Lamarckian genetic algorithm was selected with 200 runs. The interaction mode between peptides and the target protein was analyzed, and the interactions involving peptides and protein residues were determined using Pymol, version 2.1.0 software, including hydrogen bonding, π–π interactions, hydrophobic interactions, etc. Finally, the binding sites, binding energy (BE), and critical residues of peptides were determined.

#### 3.8.1. Fly Strains and Husbandry

The WT and *PINK1^B9^* mutants were generously donated by Prof. Zhuohua Zhang from the Xiangya School of Medicine, Central South University. The flies were cultured on a conventional fly medium under controlled conditions of 25 ± 1 °C and 60% relative humidity. The traditional medium for cultivating flies was prepared by boiling 90 g of yeast, 240 g of corn flour, 32 g of agar, 189 g of glucose, and 98 g of sucrose, as well as adding a preservative solution consisting of 25 mL of a 10% methyl-4-hydroxybenzoate in distilled water to make a final volume of 3.5 L. The experimental medium was prepared by supplementing formula 4–24 instant Drosophila medium (Carolina Biological Supply Company, Burlington, NC, USA) with a concentration of 0.1 mM peptide. Male individuals of both wild-type (WT) and *PINK1^B9^* flies were collected for subsequent experiments.

#### 3.8.2. Lifespan Ability

The newly hatched male WT flies and male *PINK1^B9^* mutants were collected simultaneously, followed by their division into four groups with 4 bottles per group and 40 flies per bottle. The first group was provided with WT flies and fed with formula 4–24 instant drosophila medium. The second group consisted of *PINK1^B9^* flies and was also fed with formula 4–24 instant drosophila medium. The third group comprised *PINK1^B9^* flies and received formula 4–24 instant drosophila medium supplemented with 0.1 mM peptide. The last group included *PINK1^B9^* flies and was given formula 4–24 instant drosophila medium supplemented with 1 mM resveratrol. Fresh medium was replaced every 2–3 days, while the number of flies was recorded daily.

#### 3.8.3. Climbing Ability

First, the flies were initially anesthetized using carbon dioxide, and each experimental group comprised 30 flies. The flies were subsequently transferred into vertical transparent glass tubes (15 × 1.5 cm) and acclimatized in an incubator at a temperature of 25 °C for a duration of 30 min. We gently tapped the glass to ensure that the flies settled at the bottom of the glass bottle, and their movement was recorded every 5 min for a minimum of 1 h using an infrared behavioral recorder (Yihong Technology Co., Ltd. in Wuhan, China).

#### 3.8.4. PINK1^B9^ Flies’ ATP Levels

Flies were transferred to EP tubes (30 flies in each tube), which were frozen in liquid nitrogen before separating the thoraces. The thoraces of the 5 flies were placed in 50 μL of lysis buffer, homogenized on ice, and then subjected to centrifugation at 12000 g at 4 °C for 15 min. The levels of ATP in fly thoraces were quantified using the luciferin-luciferase system by using the ATP Assay Kit.

#### 3.8.5. PINK1^B9^ Dopamine Levels

The dopamine levels were quantified in the brains of flies by adding 50 μL of citrate acetate buffer (50 mM) to an EP tube containing 30 fly brains. The sample was subsequently homogenized on ice and then centrifuged at 12,000 g for 15 min at a temperature of 4 °C. Following this, the dopamine content was determined utilizing an enzyme-linked immunosorbent assay (ELISA) kit.

## 4. Conclusions

The present study identified four peptides derived from earthworm, which have demonstrated protective activity against MPP^+^-damaged SH-SY5Y neuronal cells while exhibiting minimal cytotoxicity. It was found that GYSFTTTAER and AVFPSIVGR can improve the MPP^+^-induced MMP decline, upregulate the mRNA expression of mitochondrial autophagy factors PINK1 and Parkin, and protect nerve cells from MPP+ damage. In addition, two peptides exhibited significant PINK1 agonistic activity with EC_50_ values of 76.77 ± 1.30 and 88.50 ± 0.97 μM, respectively. Molecular docking revealed the strong H-bonding interactions between peptides (GYSFTTTAER and AVFPSIVGR) and PINK1. GYSFTTTAER and AVFPSIVGR exhibited significant neuroprotective ability by extending the lifespan, improving the climbing, and increasing the intracerebral dopamine level of *PINK1^B9^* flies. These results indicate that earthworm-derived peptides can act as PINK1 receptor agonists and help reduce mitochondrial dysfunction by promoting the autophagic removal of damaged mitochondria in PD models. This provides a scientific basis for the potential use of earthworms as novel functional foods for the treatment of neurodegenerative diseases.

## Figures and Tables

**Table 1:** PINK1 agonistic activity of earthworm peptides.

Peptide	Agonistic Rate (%) ^1^	EC_50_ (μM) ^1^
ILLIILI	17.16 ± 0.55	Nd
GYSFTTTAER	52.46 ± 0.96	76.77 ± 1.3
AVFPSIVGR	50.35 ± 0.72	88.5 ± 0.97
AGFAGDDAPR	33.45 ± 0.45	Nd
Resveratrol ^2^	91.45 ± 0.34	13.75 ± 0.58

^1^ Values are expressed as the mean ± SD (n = 3). ^2^ Positive control: resveratrol. Nd: not detected.

## Data Availability

The raw data supporting the conclusions of this article will be made available by the authors on request.

## Supplementary Materials

The following supporting information can be downloaded at https://www.mdpi.com/article/10.3390/molecules30091952/s1, Table S1: Peptides in earthworm.
