# Supplementary material for: Isolation and Characterization of Earthworm Peptides with Neuroprotective Effects in Parkinson’s Disease Models
Source: Molecules. 2025 Apr 28;30(9):1952. doi: 10.3390/molecules30091952 (PMC12074337; doi:10.3390/molecules30091952)
Supplement: Supplementary file 1 [file molecules-30-01952-s001.zip › molecules-3568130-supplementary.pdf]

Supplementary Data

Table S1. Peptides in earthworm.

Sequence	XCorr.	Abundance in spectra	Charge number	Mass	Retention time
ILLIILI	2.17	3.07E+07	2	810.6080	46.41
GYSFTTTAER	1.24	4.29E+06	2	1132.5289	25.42
AVFPSIVGR	1.15	3.86E+06	2	945.5539	43.54
AGFAGDDAPR	1.02	3.66E+06	2	976.4505	15.10
ADVPLAPQLTDAFQK	0.84	6.07E+06	2	1613.8669	70.05
ILLLKTTALA	0.83	5.87E+06	2	1056.7078	18.86
IDSGEHILTMRLTK	0.81	6.07E+06	2	1613.8669	70.05
TSGEMLGIQKM	0.74	1.00E+06	3	1210.5694	69.39
IILTLVLTVVGYA	0.72	1.58E+06	3	1374.8544	69.90
AVTGLGSVVR	0.72	6.40E+05	2	958.5698	11.15
LLLKTTALA	0.71	6.47E+07	2	943.6213	30.51
AALGMPEALIPVQ	0.69	4.60E+06	2	1309.7137	71.18
GAITMLLTDRXLNTS	0.69	6.64E+06	2	1634.8924	70.47
CNRAEFKPGQ	0.68	1.37E+08	2	1149.5529	71.06
LRSIPNKLGGVLALFAAIVV	0.68	2.31E+07	4	2051.2797	70.17
DGNGFISAAELRHVMTNLGEKLTDEE VDEMIREAD	0.66	4.79E+06	3	3904.8574	69.37
TESSKIKIGF	0.66	5.59E+07	2	1109.6239	28.33
NGXRLERIPLXVWAVLITVVLLL	0.65	2.95E+06	5	2613.6549	70.36
GVTSWGVSSGA	0.65	1.94E+06	2	1007.4693	71.55
YRPGTVALR	0.65	2.39E+06	2	1032.5922	18.86

"X" indicates that the amino acid at this position could not be clearly identified. possibly due to mass spectrometry signal interference or other reasons .
